# Beneficial Effects of Leucine Supplementation on Criteria for Sarcopenia: A Systematic Review

**DOI:** 10.3390/nu11102504

**Published:** 2019-10-17

**Authors:** Francisco M. Martínez-Arnau, Rosa Fonfría-Vivas, Omar Cauli

**Affiliations:** 1 Department of Physiotherapy, University of Valencia, 46010 Valencia, Spain; francisco.m.martinez@uv.es; 2 Frailty and Cognitive Impairment Research Group (FROG), University of Valencia, 46010 Valencia, Spain; rosa.fonfria@uv.es; 3 Department of Nursing, University of Valencia, 46010 Valencia, Spain

**Keywords:** leucine, sarcopenia, muscular mass, muscular strength, elderly

## Abstract

Objective: Treating sarcopenia remains a challenge, and nutritional interventions present promising approaches. We summarize the effects of leucine supplementation in treating older individuals with sarcopenia associated with aging or to specific disorders, and we focus on the effect of leucine supplementation on various sarcopenia criteria, e.g., muscular strength, lean mass, and physical performance. Methods: A literature search for articles related to this topic was performed on the relevant databases, e.g., the PubMed/Medline, Embase, EBSCO, Cochrane, Lilacs, and Dialnet. The identified articles were reviewed according to Preferred Reporting Items for Systematic reviews and meta-analyses (PRISMA) guidelines. Results: Of the 163 articles we consulted, 23 met our inclusion criteria, analysing the effect of leucine or leucine-enriched protein in the treatment of sarcopenia, and 13 of these studies were based on randomized and placebo-controlled trials (RCTs). In overall terms, the published results show that administration of leucine or leucine-enriched proteins (range 1.2–6 g leucine/day) is well-tolerated and significantly improves sarcopenia in elderly individuals, mainly by improving lean muscle-mass content and in this case most protocols also include vitamin D co-administration. The effect of muscular strength showed mix results, and the effect on physical performance has seldom been studied. For sarcopenia-associated with specific disorders, the most promising effects of leucine supplementation are reported for the rehabilitation of post-stroke patients and in those with liver cirrhosis. Further placebo-controlled trials will be necessary to determine the effects of leucine and to evaluate sarcopenia with the criteria recommended by official Working Groups, thereby limiting the variability of methodological issues for sarcopenia measurement across studies.

## 1. Introduction

Sarcopenia is defined as a progressive loss of muscle mass, strength, and function [1,2]. From a physiological point view, sarcopenia starts in the fifth decade of life and at a population level, proceeds at a rate of ∼ 0.8% annually. In fact, the decrease in skeletal muscle strength induced by sarcopenia, known as dynapenia, is even more precipitous, occurring at an annual rate of ∼ 2–3% [2,3] and it is estimated that more than 20% of adults aged over 65 years, and over 50% of those aged at least 80 years are sarcopenic [4]. In some pathological conditions such as liver cirrhosis [5], chronic obstructive pulmonary disease [6,7], and diabetes [8], sarcopenia can appear earlier and its progression occurs more quickly. Several studies report that the most sarcopenic individuals are at an increased risk of developing severe sarcopenia-related complications, such as an increased risk of falls [9,10], frailty [11], disability [12], and type-II diabetes [13,14], which all have a negative impact on their quality of life and premature mortality.

Within the framework of the Third National Health and Nutrition Examination Survey, Srikanthan and Karlamangla [12] have demonstrated that muscle mass is a predictor of longevity when taking into account the all-cause mortality in North American adults (aged over 55 or 65 years for men and women, respectively). One of the main ways in which sarcopenia contributes to the disease is that it alters muscular turnover and metabolism [15,16]. Moreover, older adults exhibit a decreased anabolic response to protein feeding [17,18], which is a mechanism underpinning the loss of muscle mass in sarcopenic individuals. Compared to younger adults, those aged over 65 years required ∼ 70% more protein per meal to maximally stimulate muscle protein synthesis [19]. Furthermore, at a global level, only 40% of older adults meet the recommended daily allowance for protein (0.8 g·kg^−1^·d^−1^) and 10% of older women do not even meet the estimated average requirement of 0.66 g protein·kg^−1^·d^−1^ [20,21].

One strategy to increase the muscle protein synthesis that has been investigated is the supplementation of diets with leucine, an essential branched-chain amino acid with important regulatory actions in muscles, which are at least partially mediated by the mammalian target of the rapamycin pathway [22]. Leucine modifies protein turnover in skeletal muscles, by decreasing proteolysis and by increasing protein synthesis. Physiological research reports have shown that leucine can enhance muscle protein-synthesis [23,24,25,26]. Furthermore, leucine can stimulate insulin release by pancreatic cells [27], showing that besides its beneficial effect in enhancing skeletal muscle glucose uptake, it is also an important anabolic signal in skeletal muscle.

Based on the above, administration of leucine-containing supplements is therefore a promising approach for treating sarcopenia. We took a systematic approach to analysing the current scientific evidence in this area, and to ascertaining whether the administration of leucine-containing supplements is effective in the treatment of sarcopenia. We also included interventions that used whey protein as a supplement, because these contain large amounts of leucine (approximately 13 g leucine/100 g protein [28]) and the consumption of whey protein appears to be the most effective at increasing muscle protein synthesis [29].

As a result, the main objectives of the systematic review were to (1) analyse the effects of administration of oral leucine supplements, alone or in combination with other supplements, on sarcopenia criteria, e.g., muscular mass, strength, and functional activity in older individuals; (2) evaluate the optimal leucine supplement delivery method, dose, and treatment duration in these populations; (3) identify which signs of sarcopenia most improved upon leucine supplementation; and (4) investigate the effects of leucine supplementation on secondary outcomes such as the parameters included in the integrative psychogeriatric evaluation instruments (i.e., cognitive function, ability to perform basic activities of life, mood, and quality of life).

## 2. Materials and Methods

This study was designed and developed according to the preferred reporting items for systematic reviews and meta-analysis (PRISMA) [30] guidelines, an evidence-based set of minimum items for assessing the benefits and harms of a given healthcare intervention.

### 2.1. Literature Search

We searched the literature in various electronic bibliographic databases (PubMed/Medline, Embase, EBSCO, Cochrane databases, Lilacs, and Dialnet) for all entries up until 30 March 2019, without applying any publication age limitations. The reference lists of all the relevant articles were manually cross-referenced in order to identify any additional articles. The primary search terms used for all six databases were ‘leucine’ and ‘sarcopenia’, as well as one of the following terms: ‘older’, ‘elderly’, ‘disease’ or ‘trial’.

### 2.2. Inclusion and Exclusion Criteria

To answer our research questions, we applied the following inclusion criteria to each of the considered manuscripts: (1) It was acknowledged as an original article; we included experimental studies (randomised and placebo-controlled trials) and observational studies (cohort, cross-sectional, and case-control studies); (2) it was a full text published either in English, Portuguese, or Spanish; (3) at least one of the sarcopenia criteria was measured: Muscular mass and/or muscular strength and/or physical performance; (4) a description of how sarcopenia was evaluated was defined in the methods section; (5) the concentration of leucine used in the intervention was specified or could be calculated. Studies performed in animals, case-report studies, letters to the Editor, abstracts from conferences, books, PhD theses were excluded.

### 2.3. Data Collection and Analysis

Details of the manuscripts identified in the literature search were uploaded into a web-based system, which was used to manage the screening process and remove any duplicate citations. In order to determine which studies would be included, three members of the review team independently screened the titles and abstracts of these articles and the full texts were retrieved based on our inclusion and exclusion criteria. Two reviewers independently extracted the following data from each article: The country in which the study was conducted, number of participants, age of the participants at the time of inclusion, sex of the participants, amount of leucine administered, co-administration of other substances, sarcopenia evaluation methods, and the main and secondary outcomes of the study. Any disagreement between the reviewers regarding the papers and data extracted from them was resolved by the third author.

### 2.4. Assessment of Bias

We assessed the risk of bias in each randomised and placebo-controlled study according to the Cochrane Review guidelines [31], which cover the following bias domains: Selection bias (random sequence generation and allocation concealment), performance bias (blinding of participants and personnel), detection bias (blinding the outcome assessment), attrition bias (incomplete outcome data), and reporting bias (selective reporting). The judgments were made independently by two researchers (FM-M–A and R-F-V), with any discrepancies resolved with the third author (OC).

## 3. Results

### 3.1. Summary of Identified Publications

**Figure 1:**
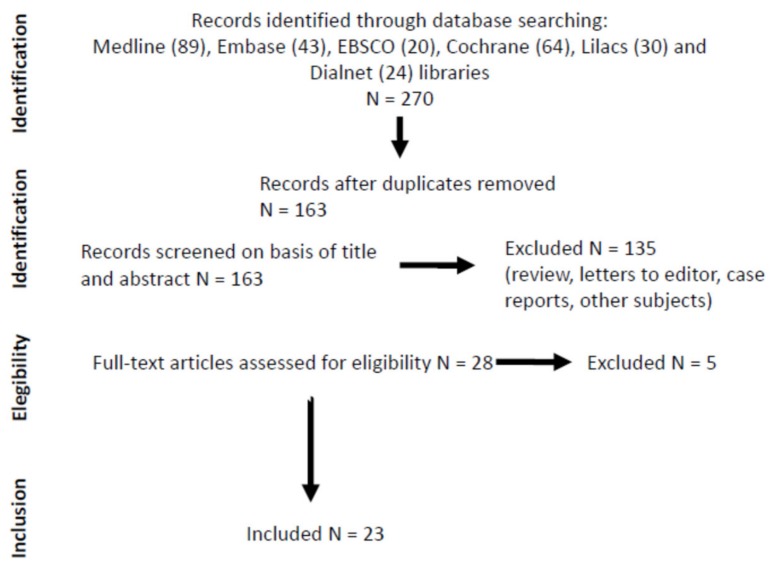
Preferred Reporting Items for Systematic reviews and meta-analyses (PRISMA) workflow for literature searches. Five of the studies [32,33,34,35,36] were not analysed in terms of the effects of sarcopenia criteria, since they were intended to study some molecular or physiological effects after short-term exposure to leucine (one to six days).

Our search strategy identified 163 studies; after eliminating duplicates and applying inclusion criteria 23 were analysed to prepare the systematic review (Figure 1). Five of the studies [32,33,34,35,36] were not analysed in terms of the effects of sarcopenia criteria, since they were intended to study some molecular or physiological effects after short term exposure to leucine (one to six days). These studies basically investigated how short-term exposure to leucine supplementation increases anabolic pathway in the muscle by measuring the rate of protein synthesis after leucine supplementation [33,34,35,36]. Kemmler et al. [32] evaluated the effect of whole-body electromyostimulation with or without short-term exposure to leucine-enriched protein supplementation in community-dwelling older men with sarcopenic obesity.

Thirteen studies were based on RCTs and were included for further assessment of risk of bias according to the Cochrane Review guidelines [31]. Two researchers independently summarised the results extracted from these articles under the following four headings: (1) The effect of leucine supplementation on sarcopenia; (2) administration, dose, duration, and eventual side effects of leucine supplementation; (3) the effects of leucine supplementation on secondary outcomes. The main characteristics of studies and details of how they measured sarcopenia-related variables are summarised in Table 1 and Table 2, respectively.

### 3.2. Effect of Leucine Supplementation on Three Sarcopenia Criteria

**Figure 2:**
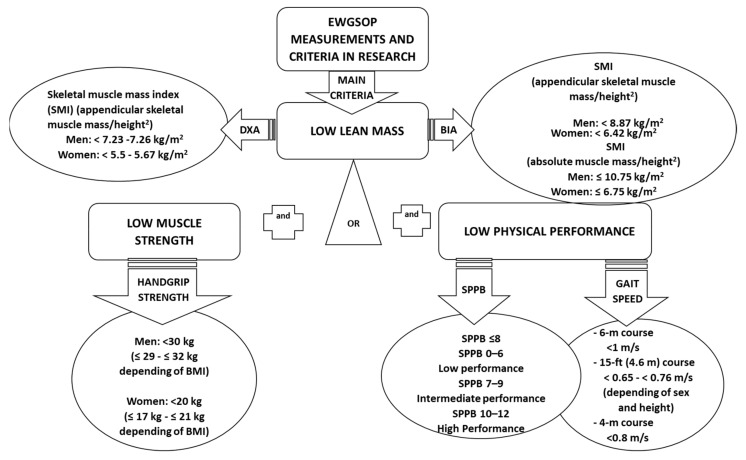
Sarcopenia criteria based on European Working Group on Sarcopenia in Older People (EWGSOP) guidelines.

Three studies were performed in Japan, two in Canada, and the others in Europe; thirteen studies classified sarcopenic individuals based on one or more accepted criteria according to specific guidelines such as those proposed by the European Working Group on Sarcopenia in Older People (EWGSOP) [1] (Figure 2) or other working groups [32,54]. The remaining studies included in the review evaluated sarcopenia by other measurements without fulfilling specific guidelines, and/or did not use cut-off points to classify sarcopenic versus non-sarcopenic individuals (Table 1). The age range of the participants was 61–87 years, and most participants were community-dwelling adults or nursing home residents of either sex. However, several studies were performed only in men [32,33,34,40,42,46,51] or in women [35,36,44].

The effects of leucine supplementation on the three sarcopenia criteria, i.e., muscle strength, lean mass, and physical performance (walking speed) are summarized in Table 2. Six of the 16 studies that evaluated muscular strength reported an improvement in muscle strength following leucine supplementation. One study found [39] a negative effect for these parameters. Neither co-supplementation with vitamin D (or other constituents) nor the leucine-administration route (among essential amino-acid (EAA) mixtures or as leucine-enriched protein preparations) had a clear effect on muscle strength gains induced by the supplementation. Ten of the 16 studies that evaluated the effect on lean mass found an improvement and all of the studies reporting co-supplementation with vitamin D had a beneficial effect on lean mass. However, some studies without vitamin D co-supplementation also showed some beneficial effects on this sarcopenia criterion [43,44]. Among the six studies that evaluated the effect on physical performance (operationalized by measuring the walking speed), three studies found a significant improvement after nutritional supplementation with leucine (with whey/casein protein or as part of an EAA mixture, with or without vitamin D).

Several studies used the 6-min walking test as a measure of low physical performance. However, even it is related to walking speed, this parameter cannot be used as a substitute for the EGSWOP criterion since the distance achieved during the 6 min walking test may be influenced by other parameters such as fatigue and cardiovascular and respiratory problems that could alter the results of the test. Other co-interventions including carnitine and creatine [41], and triglyceride (TG) [37] supplementation and electrostimulation [32] do not present more beneficial effects compared to the studies in which the nutritional intervention included leucine or whey protein with or without vitamin D. By contrast, the interventions that include a program of physical exercise [44,48,49,53], co-administered with leucine-supplementation showed more positive effects than individuals performing physical exercise with no supplementation, or only receiving nutritional intervention.

As for the effect of leucine-supplementation in sarcopenia-associated disorders, clinical trials were performed on patients with chronic obstructive pulmonary disease (COPD) [47,50], liver cirrhosis [49], type II diabetes [34], polymialgia rheumatica [39], or post-stroke patients [54]. The effect of leucine supplementation on sarcopenia criteria were weaker or absent compared to those reported in studies that did not recruit individuals with a specific pathology. The beneficial effects included an improvement in muscle strength observed in COPD patients [47] and in muscle strength and lean mass content in post-stroke patients [54] and in physical performance in cirrhotic patients when co-administered with a physical exercise program [49].

### 3.3. Administration, Dose, Duration, and Safety of Leucine Supplementation

Leucine nutritional interventions were administered alone, in an EAA mixture, or in leucine-enriched whey/casein protein (Table 2) at a dose of 1.2–6 g/day, and in nine studies leucine was co-supplemented with 85–800 IU of vitamin D per day. Thirteen studies compared the results of the intervention group(s) with a placebo-controlled group. The duration of the nutritional interventions or experiments ranged from 1 day (acute effects) to 17 weeks. In three studies leucine-containing supplements were administered only once in order to study the acute molecular effects of leucine (Table 2). Depending on the dose, acute administration of EAA mixtures or whey/casein protein stimulates albumin [35] and muscle-protein synthesis, by 20%–50% in the latter case [33,35,46]; these anabolic effects occurred to a similar extent in both sarcopenic and non-sarcopenic individuals [33].

Most studies evaluated safety based on the absence or appearance of serious side effects (e.g., nausea, diarrhoea, or gastrointestinal tract complaints) associated with the supplementation. The supplementation’s protocols tested in the studies were generally well tolerated, and none of the adverse events resulted in discontinuation of product consumption or study participation [32,33,37,40,47,48]. Only the study that used the highest dose of whey protein (160 g per day) reported some gastrointestinal complaints (early satiety, flatulence, nausea, and diarrhoea) in 48.9% of the sample [39].

### 3.4. Analysis of Moderating Factors and Subgroups

In the study by Verlaan et al. [52], appendicular muscle mass significantly increased after leucine supplementation in the individuals in the intervention group with both higher baseline protein intakes and 25(OH)D concentrations. Specifically, changes in the appendicular muscle to total body fat ratio after whey/casein protein supplementation were significant in patients aged over 69 years [39]. None of the studies involving both sexes tried to experimentally ascertain if there were any gender differences in muscle gain.

### 3.5. The Effects of Leucine Supplementation on Secondary Outcomes

As secondary outcomes, we evaluated the effect of leucine-containing supplements on other psychogeriatric evaluation parameters such as cognitive function, depression symptoms, the ability to perform activities of daily life, and quality of life. Several studies reported an improvement in cognitive functions with the administration of leucine, EAAs, or whey proteins alone [47,48,49] or with supplementation with EAAs, vitamin D, and TGs [37], although these effects may be because of the presence of the medium-chain TGs also included in the nutritional intervention used in this latter study. Rondanelli et al. [48] also reported that leucine supplementation improved patients’ functional ability to perform the activities of daily life, and others showed an improvement in the symptoms of depression [50,52]. Finally, Bauer et al. and Dal Negro et al. [38,47], but not Evans et al. [41] reported an improvement in the quality of life scores among leucine-supplemented individuals.

### 3.6. Bias Risk-Evaluation in Randomised Controlled Trials

The evaluation of risk bias in randomised and placebo-controlled clinical trials (RCT) was performed using the Cochrane guidelines mentioned above [31] in order to analyse each bias type in the 13 RCTs. A summary of bias-risk in RCTs is shown in Table 3. With regard to the selection bias, random sequence generation was unclear or high in eight studies, whereas allocation concealment was high in only three RCTs. Performance bias was clearly avoided in all the trials except for one study, in which it was unclear if the blinding of the participants and personnel to the treatments was properly performed. Avoidance of detection bias, by blinding the researchers to the assessment outcomes, was the most unmet risk of bias, and it was clearly achieved in two studies [51,52] but not mentioned in the other reports. The presence of attrition bias resulting from the collection of incomplete outcome data, was unclear in six RCTs. Reporting bias, i.e., selective reporting, was unclear in five trials: They lacked some information related to secondary outcomes or socio-demographic factors. The RCTs with the lowest risk of bias were those reported in Bauer et al. [38] and Van de Bool [50], but from a general point of view all the RCTs selected have an acceptably low level risk of biases and they are suitable for making some recommendations (see below).

## 4. Discussion

The loss of muscle mass and strength (sarcopenia) and its functional consequences such as reduced walking speed can occur to a varying degree both as a part of physiological changes during aging [55] or in some pathological conditions increasing the amount of time in bed which in itself can result in catabolism and a decrease in muscle mass [56,57] or patients with chronic diseases such as liver cirrhosis [5,58], COPD [6,7], diabetes [34] and post-stroke patients [54]. Severe sarcopenia is associated with an extremely high rate of disability but not necessarily with body-weight loss, and obese sarcopenic patients appear to have even worse outcomes [59,60,61].

The best approaches to treat sarcopenia or delay its progression over time are currently based on physical exercise and nutritional supplementation, with resistance training being the most useful tool for effectively preventing [62,63] and treating sarcopenia [64,65,66]. However, many older people are sedentary and either cannot (social barriers and family support) or do not want to exercise. In these cases, nutritional interventions remain the most promising intervention to delay the appearance of the adverse consequences of sarcopenia such as falls, mobility loss, and a bed-to-sofa lifestyle. Supplementation of the branched-chain amino acid, leucine, or leucine-enriched protein (whey/casein protein) [28,29,67] is one of the most common interventions for treating sarcopenia in older individuals, and several RCTs have been published in the last five years.

Analysis of the trials included in this systematic review showed that these nutritional interventions can improve several aspects of sarcopenia, including muscle strength, lean mass content, and walking speed (although the latter has seldom been investigated) although these effects depend on sarcopenia criteria (ranging from improvement to no effect) and the patients’ characteristics. Only one study [39] found a reduction in muscle strength after supplementation with whey protein and casein, although they did not make the reasons for this reduction clear. As possible causes, they cited an insufficient difference in protein intake between groups, a lack of a need for protein-supplementation because of already sufficient dietary protein intake, or the specific characteristics of the patients included in the sample who all had polymyalgia rheumatica. In overall terms, the results of the review suggest that the beneficial effects of leucine-supplementation are more consistent for the improvement in lean mass reported in 63% of the analysed studies, which is consistent with the physiological action of leucine in muscle metabolism.

Interestingly, the majority of interventions that found an improvement in lean mass after leucine supplementation also co-administered vitamin D as a part of the nutritional intervention. Vitamin D is increasingly recognised as playing an important role in normal muscle function, and low vitamin D status is associated with an increased risk of falls and proximal weakness [68] and these effects are more prominent in male individuals [69]. In view of these sex-dependent effects, the differential effects of leucine supplementation with or without vitamin D and the measurement of vitamin D as a biological marker of sarcopenia need further research.

In contrast, the sarcopenia criteria related to muscle strength and physical performance are not only mediated by an efficient and proper lean mass, but also by a central nervous system component which forms a neuro-muscular (motor) unit [3,70]. Improvements in neuromuscular activation precede increases in muscle mass in response to resistance training; neuromuscular activation has therefore been proposed as another measure of muscle quality [70]. The fact that in most studies there were no clear parallelism between a positive effect in lean mass improvement accompanied by a positive effect on muscle strength or physical performance confirms that the central nervous system component is necessary for restoring muscular strength in age- and diseases-related sarcopenia is less sensitive to leucine supplementation than lean mass improvement.

With regards to the effects of leucine supplementation in sarcopenia occurring in individuals with some kind of disease, an improvement in muscle strength was observed in COPD patients [47]. Muscle wasting is common in COPD, particularly among those with the emphysema-type, and associated with a high prevalence of osteoporosis, impaired exercise performance, and higher mortality risk [71]. Further RCT should address the effects of leucine supplementation on physical performance, which may definitely be relevant to patients’ ability to carry out basic activities of daily life and their quality of life.

A beneficial effect of leucine supplementation co-administered with a program of physical exercise was observed in post-stroke patients for both muscular strength and lean mass [54].

Regarding methodological issues, our analysis highlighted that different sarcopenia evaluation methods have often failed to adequately meet the criteria proposed by the international guidelines [1,72,73]. Therefore, future studies should focus on evaluating treatment effects using accepted criteria for defining sarcopenia and to select optimally the features of individuals or patients that could benefit more following an intervention consisting in leucine supplementation [72].

To the best of our knowledge, no published work has investigated the role of comorbidities or polymedication in the effects produced by leucine supplementation and as such further research in this area is clearly warranted. This would enable the selection of patients who could most benefit from supplementation, such as patients with COPD or cirrhosis, because these diseases are classically associated with sarcopenia [47,49,50]. To date, the best available evidence suggests that administration of leucine-enriched proteins such as whey/casein protein or an EAA mixture, has beneficial effects for sarcopenic individuals, is well-tolerated, and does not have any serious adverse effects.

Finally, whenever possible, a multidisciplinary approach to treating sarcopenia must be encouraged. This means that besides leucine supplementation, clinicians should prescribe a balanced diet and physical exercise to treat this condition. Finally, our analysis also provides evidence that leucine or leucine-enriched protein supplementation may also have other beneficial effects, such as improving cognitive function [47,48,49], symptoms of depression [50,52], and quality of life [38,47], which lends further support to the use of this intervention in geriatric populations.

## Figures and Tables

**Table 1:** Main characteristics and measurement of three sarcopenia criteria in the analysed studies.

	Mean Age (years)	Sample Size	Percentage of Women	Country	Characteristics of the Sample	Assessment of Sarcopenia with Recommended Tools	Lean Mass	Handgrip	Physical Performance Assessment
Abe et al. 2016 [37]	86.6 ± 4.8	38	71,05%	Japan	Nursing home residents	NO	Arm muscle area and calf circumference.	Handgrip dynamometry	Knee extension time and 10m test.
Bauer et al., 2015 [38]	77.7	380	65,52%	Netherlands registered (sample: 6 European countries)	Community-dwelling individuals	YES (EWGSOP)	Skeletal muscle mass index adjusted by BMI by BIA and DXA. (Janssen criteria) Use of cut-off values	Handgrip dynamometry	SPPB score
Bjorkman et al., 2011 [39]	69.6	47	89,36%	Finland	Non-specified residence. Polymialgia rheumatica patients	YES (EWGSOP)	Skeletal muscle mass index by DXA.	Handgrip dynamometry	Walking speed 10 m-test.
Bukhari et al., 2015 [35]	66 ± 25	16	100%	United Kingdom	Non-specified residence	YES(EWGSOP)	Appendicular muscle mass and skeletal muscle mass index by DXA (Janssen criteria)	Not determined	Not determined
Chanet et al., 2017 [40]	71 ± 4	24	0%	France	Non-specified residence	YES (EWGSOP)	Skeletal muscle mass index by DXA.	Handgrip dynamometry	SPPB score
De Vries et al., 2018 [36]	68.5	22	100%	Canada	Community-dwelling individuals	NO	Skeletal muscle mass in kg by DXA.	Knee extension strength.	Estimated physical activity by pedometer in leg and accelerometer in arm
Evans et al., 2017 [41]	57.6	42	64,29%	Canada	Community-dwelling individuals	YES (EWGSOP but only for handgrip criterion)	Skeletal muscle mass in kg by DXA.	Handgrip dynamometry.	6-min walking test
Holwerda et al., 2019 [42]	70 ± 1	41	0%	Netherlands	Community-dwelling individuals.	YES (EWGSOP but only for muscle mass and physical performance criteria))	Skeletal muscle mass index by DXA.	Knee extension and leg press strength	SPPB score
Ispoglou et al., 2016 [43]	71.5	25	56%	United Kingdom	Community-dwelling individuals	YES (EWGSOP but only for handgrip criterion)	Skeletal muscle mass in kg by DXA.	Handgrip dynamometry.	6-min walking test
Kemmler et al., 2017 [32]	77.6	100	0%	Germany	Community-dwelling individuals	YES FNIH criteria)	Skeletal muscle mass index by BIA Use of cut-off values	Handgrip dynamometry Use of cut-off values	Late life function and disability instrument (LLFDI) and physical activity index.
Kim et al., 2012 [44]	79.3	155	100%	Japan	Non-specified residence	YES (EWGSOP but only for muscle mass criterion)	Skeletal muscle mass index by BIA. Use of cut-off values	Knee extension strength Use of cut-off values	Usual walking speed. Use of cut-off values
Kirk et al., 2019 [45]	68 ± 5	46	54,3%	United Kingdom	Community-dwelling individuals	NO	Not determined.	Leg press strength	SPPB score
Kouw et al., 2017 [46]	71	48	0%	Netherlands	Non-specified residence	YES (EWGSOP but only for muscle mass criterion)	Skeletal muscle mass index by DXA.	Not determined	Not determined
Kramer et al., 2017 [33]	75	30	0%	Netherlands	Community-dwelling individuals	YES (EWGSOP criteria)	Skeletal muscle mass index by DXA. Use of cut-off values.	Handgrip dynamometry Use of cut-off values	Walking speed 4 m-test Use of cut-off values
Dal Negro et al., 2012 [47]	74.6	88	30,7%	Italy	Community-dwelling individuals. COPD patients.	NO	Skeletal muscle mass index by BIA. (Baarends criteria). Use of cut-off values	Handgrip dynamometry	Physical activity by daily steps.
Leenders et al., 2011 [34]	71 ± 1	60	0%	Netherlands	Non-specified residence. Diabetes mellitus type 2 individuals.	NO	Skeletal muscle mass in kg by DXA.	Knee extension and leg press strength	Habitual physical activity register in METs.
Rondanelli et al., 2016 [48]	80.3	130	59,23%	Italy	Nursing home residents	YES (EWGSOP but only for muscle mass criterion)	Skeletal muscle mass index by DXA Use of cut-off values	Handgrip dynamometry	Physical activity by activities of daily living.
Soriano et al., 2013 [49]	62	17	29,4%	Spain	Non-specified residence. Patients with liver cirrhosis.	NO	Arm muscle area and quadriceps perimeter.	Not determined	6-min walking test
Van den Bool et al., 2017 [50]	62.6	81	49%	Netherlands	Community-dwelling individuals. COPD patients.	NO	Skeletal muscle mass in kg by DXA.	Knee extension strength and respiratory muscle strength	6-min walking test
Verhoeven et al., 2009 [51]	71 ± 4	30	0%	Netherlands	Community-dwelling individuals.	NO	Skeletal muscle mass in kg by DXA.	Knee extension and leg press strength.	Not determined
Verlaan et al., 2018 [52]	77.7	380	65%	Netherlands registered (multicenter several countries) (18 centers)	Community-dwelling individuals	YES (EWGSOP but only for muscle mass criterion)	Skeletal muscle mass index adjusted by BMI by BIA (Janssen criteria) Use of cut-off values	Handgrip dynamometry	SPPB score.
Verreijen et al., 2015 [53]	63.4	80 I (40) C (40)	53%	Netherlands	Non-specified residence	YES (EWGSOP)	Skeletal muscle mass index by DXA.	Handgrip dynamometry.	Walking speed 4 m-test
Yoshimura et al., 2019 [54]	79.8	44	68.2%	Japan	Non-specified residence. Post-stroke patients	YES (AWGS)	Skeletal muscle mass index by BIA Use of cut-off values	Handgrip dynamometry Use of cut-off values	FIM score

AWGS: Asian Working group for Sarcopenia; BIA: Bioelectrical impedance analysis; BMI: Body mass index; DXA: Dual X-ray absorptiometry; EWGSOP: European Working Group for Sarcopenia in Older people; FIM: Functional independence measure; LLFDI: Late life function and disability instrument; SPPB: Short physical performance battery.

**Table 2:** Characteristics of leucine and placebo administration and effects on the three sarcopenia criteria.

	Leucine Dose (g/day)	Additional Supplementation Intervention Constituents (g/day)	Control Group	Treatment Duration	Effect on Muscle Strength	Effect on Lean Mass	Effect on Walking Speed
Abe 2016 [37]	1.2	Group 1: EAAs (3 g), vit-D (800IU), medium-chain TGs (6 g); Group 2: EAAs (3 g), vit-D (800IU), or long-chain TGs (6 g)	No placebo group	13 weeks	Improved	Not determined	Improved
Bauer 2015 [38]	6	Whey protein (40 g), carbohydrates (18 g), fat (6 g), vit-D (1600 IU) and mixture of vitamins, minerals and fibers	Isocaloric product without protein content	13 weeks	Not altered	Improved	Not altered
Bjorkman 2011 [39]	19.2 (Group 1) or 16.8 (Group 2) – calculated	Group 1: Whey protein (160 g) and casein (40 g) Group 2: Whey protein (40 g) and casein (160 g)	No placebo group	8 weeks (and crossover)	Decreased	Not altered	Improved
Chanet 2017 [40]	3	Protein (21 g), carbohydrates (9.5 g), fat (3 g), vit-D (20 µg) and mixture of vitamins, minerals and fibers	Flavored watery placebo drink	6 weeks	Not altered	Improved	Not altered
Evans 2017 [41]	2 (Group 1)	Group 1: Leucine (2 g), L-Carnitine (1.5 g), creatine monohydrates (3 g), Vit-D (400 IU); Group 2: L-Carnitine (1,5 g)	Placebo dissolved in orange juice	8 weeks	Not altered	Improved (only in Group 1)	Not determined
Holwerda 2019 [42]	2,8 (Group 1)	Group 1: Whey protein (20,7 g, with 2.8 g leucine), exercise (3 times/week)	Isocaloric product without protein content and exercise (3 times/week)	12 weeks	Not altered	Not altered	Not altered
Ispoglou 2016 [43]	3 (Group 1) or 6 (Group 2) – calculated	Group 1: EAA mixture (15 g); Group 2: EAA mixture leucine-enriched (15 g)	Isocaloric lactose placebo	13 weeks	Not altered	Improved (only in group 2)	Not determined
Kim 2012 [44]	2.5 (Group 1 and 3)	Group 1: EAA (6 g), exercise (2 times/week); Group 2: Exercise (2 times/week); Group 3: EAA (6 g);Group 4: Health education	Health education program	13 weeks	Improved (only in Group1)	Improved (only in Group 1)	Improved (in Groups 1 and 2)
Kirk 2019 [45]	7 - estimated	Group 1: Whey protein (1,5 g/kg/day), exercise (3 times/week) Group 2: Exercise (3 times/week)	No placebo group	16 weeks	Not altered	Not determined	Not altered
Dal Negro 2012 [47]	2.5	EAA (8 g)	Isocaloric product without protein content	12 weeks	Improved	Not altered	Not determined
Leenders 2011 [34]	7.5	Leucine alone	Wheat flour	24 weeks	Not altered	Not altered	Not determined
Rondanelli 2016 [48]	4	Whey protein (32 g with 10,9 g of EAA), Vit-D (100IU), exercise (3 times/week)	Isocaloric maltodextrine placebo (32 g)	12 weeks	Improved	Improved	Not determined
Soriano 2013 [49]	10	Group 1: Leucine and nutritional assessment; Group 2: Leucine, nutritional assessment and exercise	No placebo group	13 weeks	Not determined	Not determined	Improved
Van de Bool 2017 [50]	1.8 or 2.7 - calculated	Protein (between 18.74 and 28.11 g)	Flavored non-caloric protein-free aqueous placebo	17 weeks	Not determined	Not altered	Not determined
Verhoeven 2009 [51]	7.5	Leucine alone	Wheat flour	12 weeks	Not altered	Not altered	Not determined
Verlaan 2018 [52]	6	Whey protein (20 g), Vit-D (800 IU)	Isocaloric product without protein content	13 weeks	Not determined	Improved	Not determined
Verreijen 2015 [53]	2,8	Whey protein (20.7 g with 10.6 g of EAA), vit-D (20 µg), exercise (3 times/week)	Isocaloric product without protein content	13 weeks	Not altered	Improved	Not determined
Yoshimura 2019 [54]	3	Group 1: EAA (7.5 g), exercise (7 times/week) Group 2: Exercise (7 times/week)	No placebo group	8 weeks	Improved	Improved	Not determined

**Table 3:** Assessment of risk of bias for randomized and placebo-controlled trials (RCTs).

Bias Domain	Selection Bias		Performance Bias	Detection Bias	Attrition Bias	Reporting Bias
Source of Bias	Random Sequence Generation	Allocation Concealment	Blinding of Participants and Personnel	Blinding of Outcome Assessment	Incomplete Outcome Data	Selective Reporting
RCT Report						
Bauer et al. 2015 [38]	low	low	low	unclear	Low	low
Chanet et al. 2017 [40]	low	low	low	high	Unclear	low
Evans et al. 2017 [41]	unclear	low	low	unclear	Low	low
Holwerda et al. 2019 [42]	high	low	low	unclear	Low	unclear
Ispoglou et al. 2016 [43]	high	low	unclear	unclear	Low	unclear
Kim et al. 2012 [44]	low	low	low	unclear	Low	unclear
Dal Negro et al. 2012 [47]	high	high	low	high	Unclear	low
Leenders et al. 2011 [34]	high	high	low	unclear	Unclear	low
Rondanelli et al. 2016 [48]	unclear	low	low	unclear	Unclear	low
Van de Bool et al. 2017 [50]	low	low	low	unclear	Low	low
Verhoeven et al. 2009 [51]	high	high	low	low	Unclear	low
Verlaan et al. 2018 [52]	low	low	low	low	Unclear	unclear
Verreijen et al. 2014 [53]	low	low	low	unclear	Low	unclear
